# Efficacy of Two School-Based Interventions on Notational Ability of Bilingual Preschoolers: A Group-Randomized Trial Study

**DOI:** 10.3389/fpsyg.2021.686285

**Published:** 2021-10-15

**Authors:** Oriana Incognito, Lucia Bigozzi, Giulia Vettori, Giuliana Pinto

**Affiliations:** Department of Education, Languages, Interculture, Literature and Psychology, University of Florence, Florence, Italy

**Keywords:** bilingualism, notational skills, school-based interventions, preschool, group–randomized trial study

## Abstract

This randomized trial study aimed to analyze the efficacy of two different school-based interventions—normal preschool literacy teaching, and the PASSI intervention carried out for different durations (12 versus 30 weeks)—on notational knowledge of bilingual language-minority (BLM) preschoolers and their monolingual peers, after controlling their linguistic background and socio- economic status. A total of 251 children aged 4–5 years (M age = 4 years and 8 months; SD age = 6 months; 49% males, 51% females) were recruited from 19 classes in five preschools and randomly assigned to three groups that corresponded to different notational-focused interventions: (1) normal preschool literacy teaching (Condition 1; *n* = 47); (2) the PASSI intervention carried out for 12 weeks (Condition 2; *n* = 119); and (3) the PASSI intervention carried out for 30 weeks (Condition 3; *n* = 85). We collected two waves of data before and after the interventions regarding notational knowledge and phonological skills. Using the mixed ANOVA, we found that the PASSI intervention (both durations of 12 and 30 weeks) led to a significantly higher level of notational knowledge in BLM children and their monolingual peers. In addition, we observed that with the PASSI intervention carried out for 30 weeks, the baseline difference between BLMs and their monolingual peers was nullified. This study demonstrates that well-designed, school-based programs can benefit language-minority children by supporting their emergent notational knowledge. This paper also discusses implications for bilingual education policymaking.

## Introduction

In Western countries, linguistic heterogeneity in classrooms, especially in preschools and primary schools, is becoming increasingly prevalent, challenging educational systems to flexibly adapt their teaching practices and curricula (Dockrell et al., 2021). Given the variety of pupils’ linguistic backgrounds and home literacy experiences (Hammer and Miccio, 2006), it is essential to assess whether bilingualism and monolingualism are associated with differences in emergent literacy, a pivotal set of abilities that are developmental precursors of reading and writing (Lonigan et al., 2008; Pinto et al., 2009).

The importance of mastering emergent literacy skills for subsequent formalized literacy has been consistently documented for monolingual (Puranik et al., 2011; Pinto et al., 2017) and bilingual children (August and Shanahan, 2006; Grabe, 2010). However, the latter is an understudied population that needs to be further researched (Miller et al., 2018).

This study aimed to deepen the understanding of the emergent literacy skills of bilingual language-minority (BLM) preschoolers; specifically, it investigated whether their notational knowledge and phonological skills can be improved by different school-based interventions, compared to monolinguals, and assess which achieves the best results taking into account the durations of interventions.

Studies on the effect of bilingualism have mostly focused on formalized literacy skills and provided divergent findings. There has been little research on the effect on emergent literacy skills. Prior studies recognized that language-minority children’s basic cognitive (e.g., non-verbal reasoning, rapid naming, and phonological awareness) (Geva et al., 2000; Lipka and Siegel, 2012) and word decoding skills (Geva and Yaghoub Zadeh, 2006) are comparable to their first language (L1) peers or become comparable within the early school years (e.g., Lesaux et al., 2007). However, language-minority children show lower levels of oral language proficiency (Sammons et al., 2004), vocabulary, and reading comprehension skills (Burgoyne et al., 2011). Calvo and Bialystok (2014) found that bilingual school-age children usually show lower performance in language tasks, independent of their socio-economic status (SES). Few studies have examined the trajectories of emergent literacy skills of BLM children (e.g., phonological awareness, letter knowledge, and oral language skills) in comparison to their L1 peers. BLM children are mostly exposed and use their L1 at home; thus, their exposure to the second language (L2), also referred to as a societal language, is in the school context (Murphy, 2014). Some evidence shows that children in bilingual immigrant families, who grow up hearing a heritage language and a majority language, often reach school age with low levels of skill in both languages (Hoff, 2020). In reviewing literature concerning the emergent literacy period, results found that Spanish-speaking bilingual kindergarteners showed significantly fewer correct spellings in an invented spelling task in English language than monolingual English-speaking peers (Raynolds and Uhry, 2010). As outlined by researchers, these differences might be explained by referring to the children’s perception of English phonemes that do not exist in Spanish that in turn might lead children relying upon different skills than those used by monolingual children to spell in English. In line with these results, Bonifacci and Tobia (2017) showed that the performance of vocabulary, phonological awareness, and morphosyntactic comprehension performance in L2 (Italian-societal language) was lower in bilingual preschool children than in their monolingual peers. In contrast, Westerveld (2014) showed that bilingual Samoan/English-speaking children’s performance in expressive and receptive vocabulary, story comprehension, and letter name knowledge was higher than monolingual children; meanwhile their performance was equal in phonological awareness and story retelling quality. Other studies have provided a more complex framework of results. Print knowledge skills, oral language and measures of code-related skills (i.e., phonological awareness) were lower for Spanish-speaking BLM preschoolers, compared to their monolingual English-speaking peers; however, these differences were mitigated by the socio-economic variable (Lonigan et al., 2013). Taken together, findings on whether the condition of bilingualism negatively affects emergent literacy skills of preschoolers are divergent and fragmented, similar to the results obtained for older children.

The specialistic literature documents that the emergent literacy skills of monolingual children are modifiable through specific school-based interventions (Justice and Pullen, 2003; Pinto et al., 2018, 2019). It would also be important to verify whether BLM children are responsive to different school interventions and verify the effectiveness of different interventions for both monolingual and BLM children.

Few international studies have examined whether targeted, school-based interventions are effective for bilingual children. Even fewer are the studies aimed at identifying the optimal duration for a targeted intervention to inform teachers and school practitioners (e.g., Evans et al., 2014). Checking the length of intervention allows us to verify whether a longer duration of intervention tends to produce a larger effect size and produces more highly consistent positive results than the same intervention with a shorter duration (Gilley et al., 2015). It is important to provide school practitioners with empirical-based information about which is the efficient time period for a targeted intervention on preschoolers’ emergent literacy skills to produce the best intervention effect and to avoid tentative and unproductive actions.

As suggested by Buysse et al. (2014), a series of methodological issues challenge conclusions about the effectiveness of language and literacy curricula and instruction in preschool for bilingual children. First, there are a limited number of studies that evaluate different types of programs. Second, scarce information is available from experimental designs and methodological problems related to the small number of participants in the studies.

In a recent study, Goodrich et al. (2017) evaluated whether the intervention was differentially effective for BLM and monolingual English-speaking children regarding early literacy skills in other languages. They found that the intervention was effective but not for English-language-related, early literacy skills. In general, there were no differential effects of the intervention for BLM or monolingual children. Taken together, these findings indicate that high-quality, evidence-based instruction can improve the early literacy skills of language-minority, BLM, and monolingual children.

Thomas et al. (2020) proposed a preventive intervention aimed at stimulating oral language and emergent literacy skills in 194 French-speaking kindergarten children in Belgium with low SES and a mixed language background (i.e., the home language was different from their scholastic language). The post-test results showed an improvement in vocabulary, phonological awareness, letter knowledge, and print awareness in the experimental group compared to the control group.

The efficacy of other school-based interventions has been evaluated for BLM children by focusing on further dimensions of emergent literacy skills, such as vocabulary and storytelling. A book-reading intervention was found effective in improving L1 (i.e., Uyghur) and L2 (i.e., Mandarin) vocabulary knowledge in ethnic minority children from low SES families living in China (Chen et al., 2018). Few available results show that bilingual children’s responsiveness to interventions on emergent literacy skills is similar to that of their monolingual peers.

Overall, there are still several limitations to the current research findings on emergent literacy skills in bilinguals and on the effectiveness of their learning in the school context. The discrepancy in findings is probably due to the diversity of the bilingual children population included in the analysis (Bialystok, 2001; Murphy and Pine, 2003; Finnegan-Catibusic, 2006). The definition of bilingual children as those who use two different languages in their daily life (Grosjean, 1992) needs to be specified by considering multiple aspects (Bonifacci et al., 2018), such as the age of first exposure (simultaneous or consecutive), the learning context (familiar or scholastic), and the level of competence (balanced or dominant).

A further limitation of the generalizability of the results is the lack of attention paid to a potentially relevant variable in the literacy process—the spoken and written properties of a language, and the relationship between these two domains. As suggested by the theoretical framework of the comprehensive emergent literacy model (CELM), it is important to consider that the development of a child’s emergent literacy skills is influenced by learning contexts (e.g., school and family), as they provide learning experiences and opportunities to practice early literacy skills with the support of people around them (e.g., teachers and parents) (Rohde, 2015). There is strong evidence that the type of written transcription adopted by languages (alphabetic, syllabic, ideographic) is an important factor in reading and writing acquisition (Seymour, 2006). Instead, there is a scarcity of knowledge about emergent literacy skills’ development in BLM children who are exposed to a language that is structurally and morpho-syntactically distant from the societal language (such as, Chinese versus Italian language) in their home environment.

A further limitation of the studies of BLM children’s early literacy skills development stems from the fact that the impact of children’s SES, one of the main confounding variables in the study of bilingualism, has rarely been considered (Bonifacci et al., 2020).

The phenomenon of bilingualism is increasing in the Italian school system. Data from the Ministry of Education, University and Research (MIUR, 2018) show that in Italian kindergarten and primary schools, the incidence of children with non-Italian citizenship has reached 11.1 and 11.2%, respectively. Emergent literacy is a pivotal transition period for the development of reading and writing in monolingual and BLM children. Previous studies have identified the effectiveness of school-based interventions in improving notational knowledge in monolingual children; however, these results cannot be generalized to bilingual preschoolers, and multilingual learning environments need specific research. To the best of our knowledge, the levels of emergent literacy competence and the effectiveness of school-based interventions on emergent literacy skills in BLM preschoolers in the Italian school system are under-investigated. To fill this gap, in this longitudinal study, we aimed to investigate the efficacy of two different school-based interventions on emergent literacy skills, namely, notational knowledge in bilingual language-minority preschoolers and their monolingual peers.

This study focused on the specific population of language-minority children who are exposed to the L2-societal language predominantly at school, while they speak the L1-minority language at home. This study population reflects the current linguistic heterogeneity of the classroom population in Italian public schools (MIUR, 2017) by including bilingual language-minority children with different linguistic backgrounds, predominantly Chinese. SES was also taken into account as a covariate variable, as previous studies documented its relevance regarding children’s emergent literacy skills.

As teaching activities on notational competence have been implemented by teachers in kindergarten as part of the national curriculum, it would be useful to gain insights into implementing interventions with evidence-based positive effects in the curriculum.

The main focus of this study was to investigate whether notational knowledge and phonological awareness of BLM preschool children might benefit from different school-based interventions: (i) a normal preschool literacy teaching intervention, (ii) the PASSI intervention carried out for 12 weeks, and (iii) the PASSI intervention carried out for 30 weeks, compared to their monolingual peers. We expected a differentiated pattern of effects in bilingual and monolingual children and between the different school-based interventions proposed.

Furthermore, considering the evidence offered by the literature on the effect of SES, the moderating effect of SES and the effect of bilingual children’s linguistic background (whether their L1 was an alphabetic language or not) were also considered.

We expected that the normal preschool teaching intervention would not improve BLM or monolingual preschoolers’ phonological awareness; meanwhile, we expected that the PASSI intervention with different durations would significantly, positively improve in a targeted way BLM and monolingual preschoolers’ notational knowledge compared to their phonological awareness.

Specifically, in line with the literature, in BLM preschoolers, we expected a significant improvement in notational knowledge when practicing the targeted 30-week PASSI intervention (Condition 3), as well as when they are practicing the normal preschool literacy teaching (Condition 1) and the targeted 12-week PASSI intervention (Condition 2) (Hypothesis 1a).

In monolingual preschoolers, we expected no significant improvement in notational knowledge when practicing the normal preschool literacy teaching (Condition 1), instead we expected a significant improvement in notational knowledge when practicing the targeted 30-week PASSI intervention (Condition 3). Responsiveness to the targeted 12-week PASSI intervention (Condition 2) remained unclear for the monolingual children as this is a new research question in this field of research (Hypothesis 1b).

Finally, we expected that the 30-week PASSI intervention would reduce the initial gap in notational knowledge between monolingual and bilingual children, even though this might depend on whether the baseline task scores in notational knowledge of the participants (monolinguals and bilinguals) are the same or not (Hypothesis 2).

## Materials and Methods

### Participants

A total of 251 children aged between four and five (M age = 4 years and 8 months; SD age = 6 months; 49% males, 51% females) attending the last year of five public preschools in the central part of Italy participated in the study, which was part of a larger research study conducted on 285 children. The schools voluntarily agreed to participate in this study. School authorities, parents, and the children provided consent to participate. Children’s parents were invited to complete a questionnaire including questions about home language and socio-cultural and economic status, when their written consent to participate in the research was obtained. From the initial sample, we excluded children who had a certified developmental disorder or sensory or neurological impairment and special educational needs (about 8%), as well as those who did not complete all the tasks (either pre-or post-test; about 4%).

In this study, BLM children (34%) with Italian as their L2 were exposed to L1-minority language at home. Their Italian-speaking monolingual peers (66%) were included. The linguistic backgrounds of bilingual children were differentiated as follows: L1-minority languages included Chinese (18%), Albanian (9%), Romanian (2.4%), and other minority languages from Nigeria, South America (such as Brazil and Mexico), and India (4.6%).

All the children included in the experiment attended the last year of preschool, before their transition toward primary school. During this period, children’s writing and reading literacy is not yet formalized. The presence of bilingual children with different language backgrounds is significant in Tuscany, the Italian region where this study was conducted. Specifically, according to MIUR, in 2018, 31.2% of children attending Tuscany kindergartens were students without Italian citizenship. In line with MIUR data, the bilingual preschool children included in the sample of the present study were 33.5% of the total participants.

### Research Design

To verify the hypotheses of this study, we adopted a longitudinal research design with pre-test and post-test comparisons between BLM and monolingual preschoolers. Regarding the characteristics of our bilingual language-minority, it predominantly included children exposed to the Chinese language in their home environment.

Classrooms were randomly assigned to three groups that corresponded to different school-based interventions on notational knowledge: (1) the first group was assigned to a normal preschool literacy teaching intervention (*n* = 47) that carried out the standard activities; (2) the second group to the PASSI intervention carried out for 12 weeks (*n* = 119) targeting notational knowledge; and (3) the third group to the PASSI intervention carried out for 30 weeks (*n* = 85) targeting notational knowledge. All three groups comprised the following proportions, according to the language conditions: the first group (Condition 1: normal preschool literacy teaching intervention) consisted of 55% monolingual and 45% bilingual children; the second (Condition 2, the PASSI intervention—12-week dosage) had 65% monolingual and 35% bilingual children; and the third (Condition 3, the PASSI intervention—30-week dosage) had 75% monolingual and 25% bilingual children.

At the first data collection, Time 1 (Phase 1), the baseline task scores in notational knowledge and phonological skills in all three groups were checked.

In Phase 2, the normal preschool literacy teaching intervention (Condition 1), the targeted 12-week PASSI intervention (Condition 2), and the targeted 30-week PASSI intervention (Condition 3) were implemented.

Finally, at the second data collection, Time 2 (Phase 3; after 30 weeks for Condition 1 and 3 and after 12 weeks for Condition 2), task scores in notational knowledge and phonological skills were administered.

**FIGURE 1:**
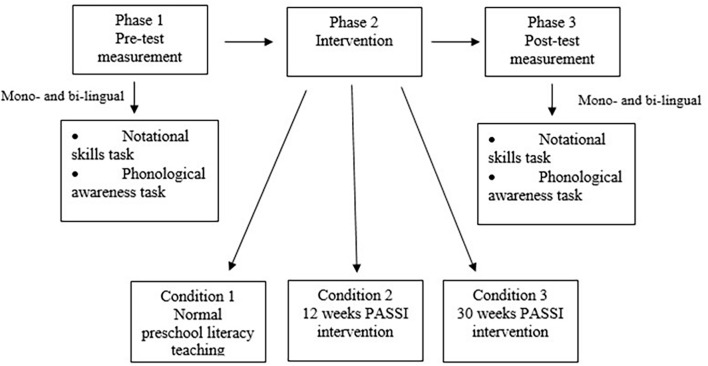
Graphical representation of the research design.

Figure 1 shows the research design adopted by this study.

### Measures

#### Socio-Economic Status

Information about family SES was collected through a parental survey attached to the informed consent form. Following the International Standard Classification of Occupations (ISCO-08, 2008) and the Standard International Socio-Economic Index of Occupational Status (ISEI; Ganzeboom et al., 1992), a socio-economic index of occupational status was calculated from collected data about parents’ occupations by combining the child’s paternal and maternal SES.

### Emergent Literacy Measures

#### Notational Skills

We used a specifically created “invented spelling task” to evaluate notational knowledge, namely conceptual knowledge of the writing system (Bigozzi et al., 2016). The task was administered individually, and each child was equipped with a pencil and a white A4 sheet of paper to perform the test, which consisted of seven items. The first item was familiarization, the children were asked “Can you write your name as you know it?.” Next, the children were asked to draw an item named by the experimenter, e.g., “apple,” “king,” and “rainbow.” In a second step, the children were asked to write down how they knew what they had drawn and then to read aloud what they had written by following it with their finger.

Items were classified on the basis of three different systems: (1) items 2 and 3: Conceptual knowledge of orthographic notation to examine how similar the children’s symbols were to conventional letters; (2) items 4 and 6: Conceptual knowledge of the orthographic variation of sound quantity to assess whether the children were aware of the numeric correspondence between sounds and symbols (one symbol per sound); and (3) items 5 and 7: Conceptual knowledge of the orthographic variation of phonemic units to verify whether the children were aware that words with similar sounds are also written similarly with small variations. Each item was given a score from 0 to 3. The average of these three scores represented children’s notational competence. The inter-judge agreement ranged from 90 to 99%. Disagreements were resolved through discussions between the two judges. Both the pre-test and post-test reliability scores were strong, with an α coefficient = 0.93 and.95, respectively.

#### Phonological Skills

The task of identification and production of sound patterns (Dowker and Pinto, 1993) was performed. The children were told, “Now I’m going to tell you a poem, which is kind of like a story but not quite. And I’d like you to make up something like it.” They were given three prompts: a rhyming poem, an alliterative poem, and a simile poem. They were then asked, “Can you make up something like this?” The order of the stimuli was counterbalanced. In the case of rhyme, it was checked to see if the child created a text containing rhymes, and in the case of alliteration, if it contained them and how many they contained. Phonological awareness was measured by aggregating the scores of the two tasks. The scores ranged from 0 to 2. Agreement between the judges was 97%, and disagreements were resolved through discussions. Both the pre-test and post-test reliability scores were good, with an α coefficient = 0.71 and.72, respectively.

### Description of the Three School-Based Interventions

As mentioned earlier, the research participants followed two different school-based interventions: (1) the first group was assigned to a normal preschool literacy teaching intervention (Condition 1), which carried out the standard scholastic activities proposed by the national guidelines; (2) a second group to the PASSI intervention carried out for 12 weeks (Condition 2) targeting notational knowledge; and (3) a third group to the PASSI intervention carried out for 30 weeks (Condition 3) targeting notational knowledge.

Fidelity of implementation was verified according to O’Donnell’s (2008) suggestions. The interventions in all three conditions were conducted by the teachers of the classes involved. Teachers applying steps received a 10-h training course on the intervention by one of the researchers. The teachers were comparable in terms of work experience: they were from the same school, had a comparable amount of teaching experience, taught the same educational curriculum, and had had the same intervention training. After the training, each teacher was supervised by a member of the research team that created PASSI. Teachers were provided with a manual, which included a detailed description of each activity, to increase the probability of fidelity of implementation. The supervisor monitored the implementation of the program through weekly meetings with the teacher, to identify significant deviations from the programmed intervention.

During the experiment, the group assigned to Condition 1 followed the usual school activities, which were based on a national curriculum established by the Ministry of Education. The school week was approximately 40 h long, and approximately three of those hours were dedicated to the improvement of emergent literacy. Specifically, according to national guidelines, kindergarten curricula must include, in their projects, activities aimed at improving children’s graphomotor, literacy-related, and sensorial skills. The main grapho-motor activities were, for example, playing with materials, transforming, and creating with the hands small and large objects, painting, coloring, and drawing patterns. Regarding the literacy activities, the children were involved in listening to and telling stories, inventing stories, illustrating stories, inventing nursery rhymes, and playing with words. Finally, examples of sensorial activities were: discriminating the basic colors; mixing them to create new colors; discriminating sounds, rhythm, high, and low pitch. However, at kindergarten, children are not usually exposed to the formal teaching of reading or spelling, which occurs in first grade. No specific teacher training was provided for these activities. However, a supervisor held meetings with the control group teachers as part of the school routine practices, during which the supervisor monitored their activity.

Groups assigned to Conditions 2 and 3 implemented the PASSI intervention (Promoting the Achievement of Sound-Sign Integration; Pinto et al., 2018), which is a specific evidence-based intervention that can improve children’s conceptual knowledge of the Italian writing system. Specifically, the intervention is aimed at promoting enhancement of the letters’ sound/symbol integration skills in preschool children. Furthermore, PASSI proposes an embedded-explicit approach aimed at improving some specific subskills of children (i.e., reflection on the graphic, symbolic, and phonological aspects of written signs), and to emphasize preschool students’ contextualized interactions with oral and written language.

The activities proposed in PASSI were designed to be similar to children’s everyday routines and offered them playful scenarios in which they could concretely use symbolic material. The activities were mainly focused on graphic, orthographic, and numerical symbols. To improve the children’s ability to graphically represent symbols, we implemented activities such as: creating shapes with cardboard or a rope; drawing shapes with chalk on the floor and then having the children walk on them; reflecting on the differences between real objects, objects in a picture, and drawn objects; reflecting on the different representations of the same object; identifying the essential traits to characterize an object through drawing; and understanding that the same graphical symbols can be assigned different meanings if represented in a different position in relation to the context. Regarding orthographic symbols, we implemented activities such as activities to familiarize the children with usual and unusual writing instruments; guessing games to discriminate written words from scribbles; activities in which the children played with letters. Finally, examples of numerical symbol activities were: nursery rhymes in which the children associated the names of the numbers with their representation; games to associate a number with symbols; activities in which the children used written numbers to discriminate positions and quantities.

Each activity lasted approximately one-and-a-half hours. The intervention suggests that the activities be conducted in the classroom once a week at the beginning of the school day. Overall, regarding condition 3, the children should work on 30 activities, 10 for each category (graphic, orthographic, and numeric signs). Regarding condition 2, the children should work on 12 activities, 4 for each category. Within each category, five activities are designed to stimulate their decoding processes and five to stimulate coding processes. Each activity consists of three tasks which vary in type and according to classroom structure. Regarding the type, PASSI proposes different kinds of activities: based on activity sheets, recycling materials, games, storytelling-based, and group discussions. Regarding classroom structure, PASSI activities can involve the entire class (for instance, discussion-based and game activities), small groups, a few students, or individuals.

### Data Analysis

According to Tabachnick and Fidell’s (2013) recommendation, before analyzing the data, the presence of univariate outliers in the phonological awareness and notational competence scores was checked. The outliers were identified and removed.

As a preliminary step, to verify the existence of linguistic influence on notational knowledge and phonological skills, at baseline task scores (Time 1), a GLM model was carried out, by considering SES level as moderator.

Regarding the aim of this study, to verify the effectiveness of the interventions on notational knowledge, mixed ANOVA with one repeated factor (two-level time) and two independent factors (two-level language condition and three-level intervention condition) was used.

The mixed ANOVA was chosen because it allows us to test for (1) differences within groups, between Time 1 and Time 2, for BLM children and monolingual children and (2) differences between groups in different conditions (the normal preschool literacy teaching, the targeted 12-week PASSI intervention and the targeted 30-week PASSI intervention) between BLM and monolingual children at Time 2. In addition, follow up comparisons were computed. In case of statistically significant differences, Cohen’s d effect size was calculated (Cohen, 1988). In agreement with Cohen’s criteria (1988), effect sizes were evaluated as negligible (*d* < 0.20), small (0.20 ≤ *d* < 0.50), medium (0.50 ≤ *d* < 0.80), or large (*d* ≥ 0.80).

As BLM children differ in transcript type (alphabetic vs. non-alphabetic), we took the next step of checking that the interventions worked on both types, using a moderation analysis.

In the same way, to verify the effectiveness of the interventions on phonological awareness, as a parallel measure of the intervention, another mixed ANOVA was used.

## Results

### Notational Skills and Phonological Awareness Preliminary Evaluation at the Pre-test

The univariate GLM was used to check the effect of preschool children’s bilingualism and SES on their notational knowledge. Table 1 presents the results.

**TABLE 1:** Effect of linguistic condition on notational skills by controlling SES.

	**SS**	**df**	***F***	***p***	**η^2^p**
Model	32.75	2	21.16	< 0.001	0.187
SES	1.12	1	1.44	0.231	0.008
Linguistic condition	30.18	1	39.00	< 0.001	0.175
Residuals	142.39	184			
Total	175.14	186			

As shown in Table 1, the condition of bilingualism explains the children’s variance in notational competence (β = −0.93; *t* = −6.25, *p* < 0.001; R^2^adj. = 0.18). Specifically, bilingualism negatively influences the results of notational competence. However, SES does not have a direct effect (main effect) on notational knowledge, nor a moderating effect on the significant relationship between linguistic background and notational knowledge. In summary, according to our first hypothesis, monolingualism is a predictor of greater success in acquisition of notational knowledge, even when children’s SES is controlled, which is not discussed further.

A second univariate GLM was performed to check for the effect of preschool children’s bilingualism, checking SES, on their phonological awareness. The model found that bilingualism does not affect phonological awareness of preschool children (β = 0.28; *t* = –1.65, *p* = n.s.; R^2^adj. = 0.02).

### Inter- and Intra-Group Differences Between Mono- and Bilingual Language-Minority and Effectiveness of School-Based Interventions on Notational Knowledge

Table 2 shows the comparison of scores (means and standard deviations) of the two groups (mono-and bilingual) at Time 1 and 2, in all intervention conditions.

**TABLE 2:** Means and standard deviation of Time 1 and Time 2 notational knowledge scores of all three experimental conditions, in each language group.

		**Time 1**	**Time 2**
Condition 1	Monolingual	2.13 (1.03)	2.34 (1.13)
	Bilingual	1.31 (0.61)	1.29 (0.56)
Condition 2	Monolingual	2.78 (0.91)	3.07 (1.10)
	Bilingual	1.60 (0.86)	2.39 (1.27)
Condition 3	Monolingual	2.08 (0.88)	3.22 (0.79)
	Bilingual	1.60 (0.63)	3.01 (1.08)

*Condition 1 refers to children who were assigned to a normal preschool literacy teaching intervention; Condition 2 refers to children who were assigned to the 12-week PASSI intervention; Condition 3 refers to children who were assigned to the 30-week PASSI intervention.*

Regarding our first hypothesis, in which we expected differences within groups, between Time 1 and Time 2, for BLM children and monolingual children, the mixed ANOVA with a Greenhouse-Geisser correction determined that means of notational knowledge differed statistically significantly between time points [within group the main effect of time: *F*(1, 35.87) = 94.13, η^2^ = 0.29, *p* < 0.001 interaction of time × linguistic background × intervention condition: *F*(5, 24.99) = 13.12, η^2^ = 0.22, *p* < 0.001]. Specifically, follow up comparison within the group showed that BLM notational knowledge scores improved significantly only in the targeted 12-week PASSI intervention (condition 2) and the targeted 30-week PASSI intervention (condition 3), but not in the normal preschool literacy teaching (condition 1) (Hypothesis 1a). In the same way, follow up comparison within the group showed that monolinguals’ notational knowledge scores improved significantly only in the targeted 12-week PASSI intervention (condition 2) and the targeted 30-week PASSI intervention (condition 3), but not in the normal preschool literacy teaching (condition 1) (Hypothesis 1b).

Regarding our second hypothesis, in which we expected differences between groups in different conditions (the normal preschool literacy teaching, the targeted 12-week PASSI intervention and the targeted 30-week PASSI intervention) between BLM and monolingual children at Time 2, the mixed ANOVA with a Greenhouse-Geisser correction determined that means of notational knowledge differed statistically significantly also between group (main effect of linguistic background: *F*(1, 47.74) = 34.08, η^2^ = 0.13, *p* < 0.001; main effect of intervention condition: *F*(2, 17.08) = 12.20, η^2^ = 0.10, *p* < 0.001; interaction of linguistic background × intervention condition: *F*(5, 108.88) = 15.55, η^2^ = 0.25, *p* < 0.001).

Specifically, Bonferroni *post hoc* showed that for Condition 1, at Time 2, the results confirm the differences in notational knowledge (*d* = 1.21; monolingual > BLM children); in Condition 2, at Time 2, there were significant differences that existed at Time 1 regarding notational knowledge (*d* = 0.55; monolingual > BLM children); however, in Condition 3, which initially had differences, the gaps were no longer present (*p* = n.s.).

**FIGURE 2:**
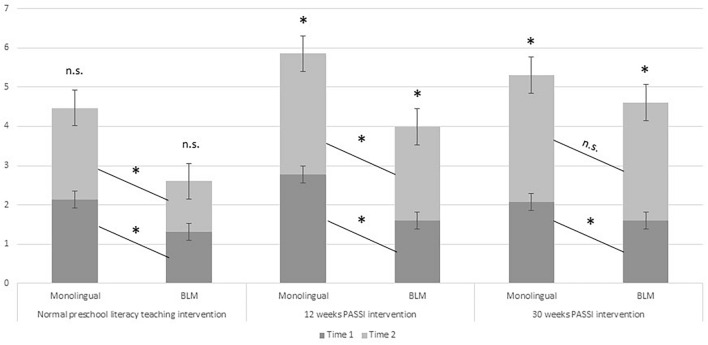
Differences in notational knowledge scores within and between groups for each intervention condition. * Refers to statistically significant differences between and within groups.

This analysis allowed us to test the differential effectiveness of different types of school interventions and their dosage between monolingual and BLM children (see Figure 2).

As previously mentioned, the group of bilingual children was differentiated based on their linguistic background: BLM children with an L1-minority language with alphabetic transcription vs. BLM children with a non-alphabetic transcription.

To test whether the different linguistic backgrounds of BLM children moderated the influence of the three school-based interventions on notational knowledge, we used GLM for interaction analyses. The results show that the direct effect of the three school-based interventions on BLM children’s notational knowledge is not moderated by their linguistic background (Table 3).

**TABLE 3:** Moderation effect of transcription type (alphabetic vs non-alphabetic) in the direct influence of interventions on bilingual children’s notational knowledge.

	**SS**	**df**	***F***	***p***	**η^2^p**
Model	40.584	5	8.054	< 0.001	0.386
Interventions	20.655	2	10.247	< 0.001	0.243
Transcription type	7.614	1	7.555	0.008	0.106
Interventions × Transcription type	0.745	2	0.370	0.692	0.011
Residuals	64.500	64			
Total	105.083	69			

*Interventions refer to the three experimental conditions; transcription type refers to the two types of transcription in bilingual children’s groups.*

### Inter- and Intra-Group Differences Between Mono- and Bilingual Language-Minority and Effectiveness of School-Based Interventions on Phonological Awareness

Table 4 shows the comparison of scores (means and standard deviations) of the two groups (mono-and bilingual) at Time 1 and 2, in all intervention conditions.

**TABLE 4:** Means and standard deviation of Time 1 and Time 2 of phonological awareness scores of all three experimental conditions, in each language group.

		**Time 1**	**Time 2**
Condition 1	Monolingual	0.28 (0.61)	0.32 (0.56)
	Bilingual	0.62 (0.80)	0.38 (0.60)
Condition 2	Monolingual	0.27 (0.52)	0.37 (0.63)
	Bilingual	0.32 (0.61)	0.43 (0.79)
Condition 3	Monolingual	0.27 (0.52)	0.52 (0.79)
	Bilingual	0.12 (0.50)	0.19 (0.54)

*Condition 1 refers to children who were assigned to a normal preschool literacy teaching intervention; Condition 2 refers to children who were assigned to the 12-week PASSI intervention; Condition 3 refers to children who were assigned to the 30-week PASSI intervention.*

The mixed ANOVA with a Greenhouse-Geisser correction determined that means of phonological awareness did not differ significantly between time points (within group main effect of time: *F*(1, 0.22) = 0.81, *p* = n.s.; interaction of time × linguistic background × intervention condition: *F*(5, 1.88) = 1.38, *p* = n.s.) or between group [main effect of linguistic background: *F*(1, 0.01) = 0.01, *p* = n.s.; main effect of intervention condition: *F*(2, 0.38) = 0.76, *p* = n.s.; interaction of linguistic background × intervention condition: *F*(5, 2.65) = 1.06, *p* = n.s.]. Comparison within the group showed that both monolingual and BLM notational knowledge scores did not improve under any of the intervention conditions. Between groups, pairwise comparisons showed no statistically significant differences.

## Discussion

This longitudinal study tested the efficacy of two different school-based interventions—normal preschool literacy teaching and the PASSI intervention carried out for different durations (12 vs. 30 weeks)—in enhancing the notational knowledge of BLM preschoolers and their monolingual peers.

The preliminary results of the comparison of baseline notational task scores show that the condition of language-minority preschoolers is associated with a disadvantage in notational knowledge compared to their monolingual peers, independent of their SES. This novel result suggests that the disadvantages in emergent literacy in BLM children can be found earlier than prior studies have documented in older children (e.g., Sammons et al., 2004; Burgoyne et al., 2011; Calvo and Bialystok, 2014). From a methodological point of view, an equal starting condition at the baseline task score was not an experimentally induced condition; on the contrary, it assured ecological validity of the research. Specifically, our preliminary results revealed that BLM preschoolers in our sample had lower notational knowledge than monolinguals. This result might be explained by referring to the differences existing between the two writing systems that BLM preschoolers were learning, given that their L2-Italian has an alphabetic orthography, meanwhile their L1-Chinese has a non-alphabetic morphosyllabic orthography (see, Verhoeven and Perfetti, 2021 for a systematic analysis of writing systems). The alphabetical transcription system of the Italian language (L1 for monolingual children and L2-societal language for bilingual children in our sample), as well as of other minority languages (e.g., Albanian, Romanian) consists of a varying number of letters that have a one-to-one correspondence with the phonemes (Pinto et al., 2018). Italian language has a transparent sound-symbol matching, as in other orthographic systems (e.g., Greek), in contrast to other languages where sound-symbol matching is more ambiguous (e.g., English). Research on children’s understanding of the notational systems shows that bilingual preschoolers must acquire appropriate representations for each language (Westerveld, 2014) with which they are familiarized at home and at school. The predominant L1-minority language, that in our sample is Chinese, develops in a different and more complex way than other language systems (Chan et al., 2008). Chinese is a logographic writing system in which written symbols or characters represent lexical morphemes. The structure of the majority of characters (80–90%) is composed of two components: a radical, that gives a meaning clue, and a phonetic component, that offers a pronunciation clue (Shu and Anderson, 1997). Each character is a syllable and a morpheme. There are no polysyllabic words, but morphemes are regularly combined to form compounds. Verbs do not change forms according to tense or plurality of the subject, and tense is encoded by completely different characters. The results of other studies on bilingualism have shown that although children recognize that their L1 and L2 writing systems are different, exposure to reading and writing at a young age helps in developing a deeper level of basic understanding of language rules and knowledge of sound-symbol correspondence, which can serve as a foundation for literacy development in other languages (Buckwalter and Gloria Lo, 2002).

A different pattern emerged from the preliminary analysis of the baseline of phonological tasks scores (Time 1). BLM children’s phonological skills, in fact, are similar to those of their monolingual peers, independent of their SES. This result is in line with other studies that suggest no significant differences in phonological awareness between monolingual and bilingual preschool children (Hamilton and Gillon, 2006). BLM preschoolers in our sample reach an equal level of phonological awareness in L2 as their monolingual peers, as revealed by the preliminary analysis. Reasonably, it is possible to expect that any possible initial difficulties in L2 phonological competence could easily be compensated by children’s constant and incidental involvement in paying attention to diverse sounds and mastering a broader set of sounds. Also, some scholars argue that different patterns of phonological knowledge can be transferred from one language to another (Bialystok, 2007).

Regarding the aim to investigate whether notational knowledge of BLM preschool children might differently benefit from two diverse school-based interventions or dosage of the same intervention (i.e., PASSI), as expected, the results at Time 2 showed differentiated effects. Our results show that within BLM preschoolers and monolinguals peers, the implementation of normal preschool literacy teaching intervention did not show significant improvements in notational knowledge between the pre- and post-test (Time 1 versus Time 2). The effectiveness of normal preschool teaching was small and did not reach significance, probably because of the occasional and incidental structure of this type of intervention. This result suggests that the simple possibility of having access to signs and written artifacts provided by the environment, generic activities are not effective and that sound-sign matching and writing systems cannot be spontaneously learned; rather, they need to be stimulated by teaching adults implementing targeted notational-focused interventions focused on the properties of the writing system, the forms and types of symbols, and the matching sound-symbol rules, which are highly conventional and arbitrary. Thus, targeted scaffolding and educational routines (Bruner, 1985; Tamis-LeMonda et al., 2019) are necessary.

Furthermore, our results show that within BLM and monolingual preschool children the PASSI intervention carried out for 12 weeks and the PASSI intervention carried out for 30 weeks were effective in improving notational knowledge. Teachers implemented PASSI to train children’s metalinguistic reflection on the writing system in instructional scaffolding. Repeated and structured activities involved children in producing invented forms of writing. Activities were proposed to familiarize the child with the tools (common and unusual) useful for writing: games of recognition of written words, to distinguish from scribbles; games with letters; search for letters within more complex configurations; reading of symbols; writing messages, letters, postcards written as successful children; discussion on the various ways of writing letters; game of dictating to the teacher of long and short words; common writing of captions under drawings. For these characteristics, the PASSI intervention was able to work in the zone of proximal development (ZPD; Vygotsky, 1978). According to the Vygotskian concept, ZPD refers to the developmental space that can enhance a child’s skill if supported by the teacher. Regarding the efficacy of the brief PASSI intervention (12-week short-term intervention), it was found that in BLM preschoolers and monolingual peers, it was linked to a consequent reduction in school time spent on training these skills and resources in the preschool teaching curriculum.

A further element to evaluate the effectiveness of the proposed school-based intervention was carried out by checking whether each intervention produces improvements as well as allows the closing of the initial gap in notational knowledge between BLM preschoolers and monolingual peers. This in-depth exploration allowed us to better understand the impact of the school context on emergent literacy development (Rohde, 2015). Specifically, it provided empirical information about which intervention is more effective in reducing initial disadvantages in emergent literacy skills with the aim of promoting equality in education between BLM children and their monolingual peers. The results of notational-skill comparisons between BLM and monolingual peers after each intervention showed that although the targeted 12-week PASSI intervention significantly improved BLM preschoolers’ performances in notational knowledge, they were still underachieving compared to their monolingual peers. In contrast, after the targeted 30-week PASSI intervention, BLM children significantly improved their performance in notational knowledge, which reached a score equal to that obtained by their monolingual peers. The results highlighted the usefulness of the protracted scaffolding time of the targeted 30-week PASSI intervention in enhancing the automatization of notational processes in BLM preschoolers, who are specifically disadvantaged in notational knowledge. Furthermore, it is possible to hypothesize that a prolonged dosage of intervention on notational knowledge might increase children’s notational knowledge, motivation in participating at reading and writing practices in schools and family contexts, as well as L1-L2 transfer of language-specific information (Goodrich et al., 2016). Due to the implementation of the proposed school-based long-term PASSI intervention, the educational context succeeded in enhancing preschoolers’ notational knowledge; it also succeeded in closing the initial performance gap, establishing a condition of equity in skills in view of future formalized schooling. In the presence of severe discrepancies in notational knowledge of BLM preschoolers, therefore, teachers may choose to implement the 30-week targeted intervention that achieves the best results. The specific strong effect of the targeted long-term PASSI intervention on notational knowledge of BLM preschoolers is reflected in them reaching a level similar to monolinguals’ emergent literacy measures during the second data collection, after the intervention. This significant improvement in BLM preschoolers and monolingual peers suggests that the targeted long-term PASSI intervention on notational knowledge significantly improved the notational knowledge of the BLM group.

An additional finding is that none of the proposed school-based interventions recorded a significant improvement in preschoolers’ phonological competence. A bi-directional developmental relationship between literacy and phonological awareness was confirmed in the literature (Landerl et al., 2019). Phonological awareness refers to children’s ability to identify and manipulate sounds, independently of their written forms, linked to later reading and writing acquisitions (Lonigan et al., 2000). The results of this study show the natural maturational improvement of phonological awareness between Time 1 and Time 2, however, the actual prompting of the school-based intervention proposed did not significantly improve BLM or monolingual preschoolers’ phonological awareness. However, it is important to consider that phonological competence concurs to notational competence involving children’s ability to distinguish between different sounds that compose a written word. In this respect, our results shed light on the efficacy of the targeted school-based PASSI interventions with different dosage (12 or 30 weeks) in improving the subcomponents of notational knowledge, including the phonological aspects of written signs. The oral level of phonological competence needs to be specifically supported in the preschool period through evidence-based activities, without anticipating formalized schooling.

Overall, the results show that bilinguals benefit from evidence-based educational practices, aggregative practices between peers and teachers at school, in addition to what they experience in the family context. The results of this study have practical implications because of the relevance of children’s notational knowledge for further reading and writing development. As already known, the improvement of emergent literacy skills in BLM children has direct, positive effects on general cognitive and language abilities (e.g., text comprehension) (Bialystok and Craik, 2010), of which literacy is a tool (e.g., for the studying activity). Also, it has an indirect positive impact on the socio-relational skills necessary for participating in school group activities, which appear compromised in BLM children (Plenty and Jonsson, 2017; Hoff, 2020). Our findings extend previous research on the effectiveness of the PASSI intervention to bilingual children, demonstrating not only that these children may benefit from this early intervention but also that this may significantly reduce their (pre)literacy gap and disadvantage. In addition, the results inform about the optimal duration of a targeted PASSI intervention to significantly promote children’s notational skills. The effects of the long-term PASSI intervention in promoting education equality are found across the different SES levels of BLM children and their monolingual peers; thus, this intervention can significantly balance the level of notational knowledge of children with low SES with that of their peers. Furthermore, the PASSI intervention (both 12- and 30-week dosage) proved to improve BLM children’s notational knowledge, independently of the properties of their writing systems (i.e., alphabetic transcription vs. non-alphabetic transcription). The result that BLM children’s notational performances are similar across different L1-minority language backgrounds emphasizes the importance of educational interventions on early implementation of notational knowledge. If offered in the preschool period, they seem to be able to prevent the difficulties that bilinguals may experience if their writing systems in L1 differ from those in L2. These findings highlight the relevance of preschool years as a rich area of potential development, particularly promising for interventions to prevent the onset of disparities in bilingual children’s transition to literacy.

### Limitations and Future Research

A limitation of this research is the measurement of SES exclusively in terms of parental occupation. In future studies, it would be interesting to take into account a more comprehensive measurement of SES, including parental education level and home literacy practices. This would inform about the weight that those factors exert on literacy development, directly, indirectly, or in interaction. In future studies, it would be useful to obtain follow-up data to evaluate the long-term longitudinal stability of the efficiency of interventions, as well as their impact on later learning of reading and writing. Furthermore, it would be interesting to see if interventions might be extended to other children’s language backgrounds with different characteristics of their writing system.

Finally, the limitation of the lacking of more rigorous fidelity checks should be filled in future studies (see, e.g., Graham and Alves, 2021).

## Data Availability Statement

The raw data supporting the conclusion of this article will be made available by the authors, without undue reservation.

## Ethics Statement

The studies involving human participants were reviewed and approved by University of Florence Ethics Committee. Written informed consent to participate in this study was provided by the participants’ legal guardian/next of kin.

## Author Contributions

All authors listed have made a substantial, direct and intellectual contribution to the work, and approved it for publication.

## Conflict of Interest

The authors declare that the research was conducted in the absence of any commercial or financial relationships that could be construed as a potential conflict of interest.

## Publisher’s Note

All claims expressed in this article are solely those of the authors and do not necessarily represent those of their affiliated organizations, or those of the publisher, the editors and the reviewers. Any product that may be evaluated in this article, or claim that may be made by its manufacturer, is not guaranteed or endorsed by the publisher.
